# Lenalidomide reduces microglial activation and behavioral deficits in a transgenic model of Parkinson’s disease

**DOI:** 10.1186/s12974-015-0320-x

**Published:** 2015-05-14

**Authors:** Elvira Valera, Michael Mante, Scott Anderson, Edward Rockenstein, Eliezer Masliah

**Affiliations:** Department of Neurosciences, University of California San Diego, 9500 Gilman Drive, La Jolla, CA 92093-0624 USA; Department of Pathology, University of California San Diego, 9500 Gilman Drive, La Jolla, CA 92093-0624 USA

**Keywords:** Lenalidomide, Parkinson’s disease, Neuroinflammation, Microgliosis, Cytokines

## Abstract

**Background:**

Parkinson’s disease (PD) is one of the most common causes of dementia and motor deficits in the elderly. PD is characterized by the abnormal accumulation of the synaptic protein alpha-synuclein (α-syn) and degeneration of dopaminergic neurons in substantia nigra, which leads to neurodegeneration and neuroinflammation. Currently, there are no disease modifying alternatives for PD; however, targeting neuroinflammation might be a viable option for reducing motor deficits and neurodegeneration. Lenalidomide is a thalidomide derivative designed for reduced toxicity and increased immunomodulatory properties. Lenalidomide has shown protective effects in an animal model of amyotrophic lateral sclerosis, and its mechanism of action involves modulation of cytokine production and inhibition of NF-κB signaling.

**Methods:**

In order to assess the effect of lenalidomide in an animal model of PD, mThy1-α-syn transgenic mice were treated with lenalidomide or the parent molecule thalidomide at 100 mg/kg for 4 weeks.

**Results:**

Lenalidomide reduced motor behavioral deficits and ameliorated dopaminergic fiber loss in the striatum. This protective action was accompanied by a reduction in microgliosis both in striatum and hippocampus. Central expression of pro-inflammatory cytokines was diminished in lenalidomide-treated transgenic animals, together with reduction in NF-κB activation.

**Conclusion:**

These results support the therapeutic potential of lenalidomide for reducing maladaptive neuroinflammation in PD and related neuropathologies.

**Electronic supplementary material:**

The online version of this article (doi:10.1186/s12974-015-0320-x) contains supplementary material, which is available to authorized users.

## Background

Disorders with parkinsonism and dementia affect over ten million people worldwide. Jointly, this heterogeneous group of disorders is known as Lewy body diseases [1–3] and includes idiopathic Parkinson’s disease (PD), PD dementia, and dementia with Lewy bodies. Neuropathologically, these disorders are characterized by accumulation of protease-resistant alpha-synuclein (α-syn) in synapses and axons, formation of neuronal inclusions, known as Lewy bodies, and degeneration of selected neuronal populations in the neocortex, limbic, and striato-nigral systems, accompanied with neuroinflammation [4–6]. α-syn is a 14 kDa neuronal protein involved in synaptic transmission and vesicle release [7, 8], and increasing evidence supports the notion that progressive accumulation of α-syn plays a central role in the pathogenesis of PD. The mechanisms through which α-syn triggers neurodegeneration are not completely understood; however, several lines of investigation support the possibility that formation of toxic α-syn aggregates might be important [9, 10]. Moreover, recent studies suggest that under pathological circumstances, toxic α-syn aggregates can be secreted [11–13]. These extracellular α-syn aggregates can then transfer from neuron to neuron [14] or from neuron to glial cells [15, 16], where they trigger and activate pro-inflammatory pathways and exacerbate the neurodegenerative process.

In PD and animal models, activated microglia and astroglia with increased tumor necrosis factor α (TNFα) has been described [17]. Toll-like receptors (TLRs) are elevated in microglia [18], and α-syn oligomers activate microglia promoting the release of TNFα and interleukin (IL)-6 in a TLR2-dependent manner [18–20]. The downstream signaling mechanisms leading to neuroinflammation are not fully understood, but recent evidence supports a role for NF-κB activation [21]. Therefore, given the potential toxicity of α-syn aggregates via pro-inflammatory pathways, therapeutic approaches for PD might involve both reducing the levels of α-syn and modulating neuroinflammatory cascades. Our hypothesis is that compounds that inhibit NF-κB signaling might attenuate neuroinflammation and degeneration in a α-syn transgenic (tg) model of PD. In this sense, thalidomide and its derivatives have been shown to protect in several inflammatory and autoimmune disorders by inhibiting the production of TNFα and other pro-inflammatory cytokines [22–24], and by blocking NF-κB signaling [21].

In this context, we evaluated the efficacy of lenalidomide and its parent molecule thalidomide at reducing neuroinflammation and neuropathology in the mThy1-α-syn tg model of PD. While lenalidomide did not reduce α-syn load, it did ameliorate behavioral deficits, dopaminergic fiber loss, and microgliosis. This effect was accompanied with diminished expression of TNFα and other pro-inflammatory cytokines and increased expression of IL-10, fractalkine, and IL-13, which have anti-inflammatory properties. These results support the hypothesis that lenalidomide reduces neuroinflammation and might be of use in PD and related disorders.

## Methods

### Animal treatments

Mice expressing human α-syn under the control of the murine Thy1 promoter (mThy1-α-syn tg) were generated as previously described [25]. A total of 38 9-month-old mice were used in this study. The mThy1-α-syn tg mice and their non-tg littermates were treated for 5 weeks with either vehicle (0.5 % methocellulose), lenalidomide, or thalidomide (100 mg/kg). Neither lenalidomide nor thalidomide had a significant effect on the parameters analyzed in non-tg animals, and results are not shown for the sake of clarity. Vehicle or drugs were administered via gavage 5 times a week in a 5 ml/kg volume. Solutions were made fresh weekly. All animal procedures were approved by the UCSD Institutional Animal Care and Use Committee.

### Total activity and round beam test

The behavioral assessment of the animals was performed using the open field and the round beam tests. As previously described [26], the total activity test was conducted for four trials each day for a period of 4 days. Total activity was calculated as total beam breaks in 10 min, and thigmotaxis was calculated as the percentage of time spent in the periphery.

The round beam test allows for the assessment of gait and balance impairments over a round beam placed horizontally [26]. Three consecutive trials of 1 min each were run in 1 day. The total distance traveled forward and the number of foot slippages were recorded. Speed on the beam is calculated as ‘distance traveled/time,’ and errors on the beam are calculated as ‘foot slips/distance traveled’.

### Immunohistochemical analysis

After behavioral analysis, mice were sacrificed under anesthesia following National Institutes of Health (NIH) guidelines for the human treatment of animals, and brains were removed. The right hemibrain was fixed by immersion in 4 % paraformaldehyde in PBS pH 7.4 and serially sectioned at 40 μm with a Vibratome apparatus (Leica) for subsequent analysis. The left hemibrain was stored at −80 °C for biochemical analysis, and further processed for either quantitative real-time polymerase chain reaction (qPCR) or protein analysis.

Vibratome sections were immunolabeled overnight with antibodies against α-syn (Sigma, 1:250), glial fibrillary acidic protein (GFAP) (Millipore, 1:500), ionized calcium-binding adapter molecule 1 (Iba1) (Wako, 1:2000), or tyrosine hydroxylase (TH) (Millipore, 1:500) followed by incubation with species-appropriate secondary antibodies (Vector Laboratories). Sections were reacted with 3,3′-diaminobenzidine (Vector Laboratories) and imaged on an Olympus BX41 microscope. A minimum of 100 cells were counted per animal, and cell counts are expressed as the average number of positive cells per field (230 μm × 184 μm). Quantification of GFAP, Iba1, and TH staining was performed by obtaining optical density measurements using the Image Quant 1.43 program (NIH) and corrected against background signal levels.

### Quantitative real-time PCR analysis

Total RNA was extracted from the mouse anterior hemibrain using a Qiagen RNeasy kit and following the instructions of the manufacturer. RNA (0.5 μg) per sample were used for reverse transcription to cDNA using a High capacity cDNA reverse transcription kit (Applied Biosystems). qPCR was performed using TaqMan Fast Advanced Master Mix and the appropriate TaqMan primers (Life Technologies). qPCR reactions were run in an StepOnePlus Real-Time PCR system, and ΔΔCt calculations were made using StepOne software (Applied Biosystems).

### Immunoblot analysis and mouse cytokine array

Protein homogenates were prepared from the mouse posterior hemibrain. Briefly, frozen samples were sonicated in homogenization buffer (HEPES 1 mM, benzamidine 5 mM, 2-mercaptoethanol 2 mM, EDTA 3 mM, MgSO_4_ 0.5 mM, NaN_3_ 0.05 %, protease inhibitor cocktail set III 1:100, phosphatase inhibitor cocktail set II 1:100) and ultracentrifuged at 100,000 rpm for 1 h to obtain cytosolic (soluble) and particulate (insoluble, membrane bound) fractions. Twenty micrograms of protein from the cytosolic or particulate fractions were loaded onto 4–12 % Bis-Tris SDS-PAGE gels (Invitrogen) and transferred onto Immobilon membranes. After overnight incubation with antibodies against total α-syn (Millipore), NF-κB p65 (Santa Cruz), CX3CL1 (Santa Cruz), or TNFα (Santa Cruz), membranes were incubated in HRP-linked secondary antibody (American Qualex), reacted with ECL Western blotting substrate (Perkin Elmer), and developed in a VersaDoc gel-imaging machine (BioRad). Immunoblotting images were analyzed using Quantity One software (BioRad).

The relative levels of 40 different mouse cytokines were analyzed using a Mouse cytokine panel array (R&D Systems) in 400 μg of protein from the cytosolic fraction of brain tissue homogenates (*n* = 4 per condition), following the instructions of the supplier.

### BV-2 cell culture

The murine microglial cell line BV-2 was cultured as previously described [27]. For protein extraction and western blotting, cells were plated onto 12-well plates at a cell density of 1.15 × 10^5^ cells per cm^2^. Human α-syn oligomers were prepared as previously described [28]. For *in vitro* treatments, the concentrations used were as follows: human α-syn aggregates, 5 μM; lenalidomide, 250 μg/ml; and thalidomide, 250 μg/ml. Controls included vehicle-only-treated cells and cells treated with Kdo_2_-Lipid A 100 ng/ml (Cayman Chemicals) (not shown). For protein analysis, samples were centrifuged at 5000×*g* for 5 min to obtain cytosolic and nuclear fractions. Immunobloting was performed as described above, using antibodies against NF-κB p65 (C-20, Santa Cruz), TNFα (Santa Cruz), and actin (Millipore). qPCR analysis was performed following the same protocol for mouse samples.

### Statistical analysis

Differences between groups (*n* = 5–7) were tested using Student’s *t* test or one-way analysis of variance (ANOVA) with Dunnett’s post hoc test. For *in vitro* assays, all conditions were assayed in duplicate and repeated in at least two separated experiments. All results are expressed as mean ± SEM. The following legends were used for denoting significance: *, #*p* < 0.05; **, ##*p* < 0.01; ***, ###*p* < 0.001.

## Results

### Lenalidomide reduced behavioral deficits and dopaminergic fiber loss in mThy1-α-syn tg mice

**Fig. 1:**
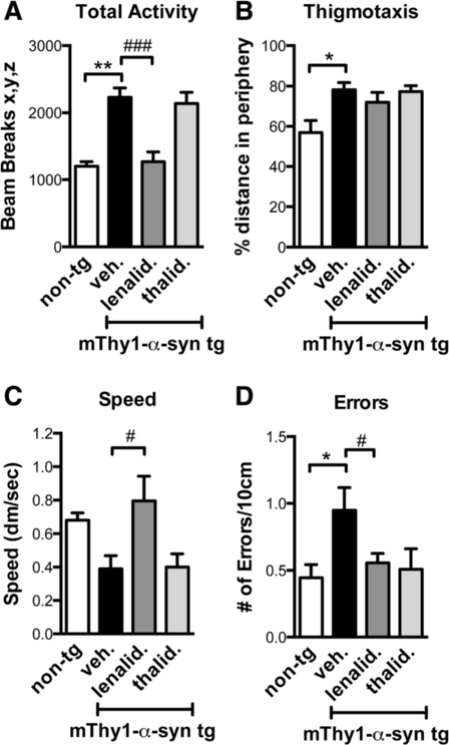
Lenalidomide reduces motor behavioral deficits in mThy1-α-syn tg mice. Non-tg and tg animals treated with either vehicle, lenalidomide, or thalidomide were analyzed in the open field and round beam tests. **a** Total activity, measured as total beam breaks. **b** Thigmotaxis, measured as the percentage of time spent in the periphery. **c** Speed in the round beam test, measure as decimeters travelled per second. **d** Number of slippages (errors) per 10 cm in the round beam test. Error bars represent ± SEM. *, #*p* < 0.05; **p < 0.01; ###*p* < 0.001

In this study, we focused on the mThy1-α-syn tg mouse model of PD, which expresses α-syn under the control of the murine Thy1 neuronal promoter and recapitulates behavioral and neuropathological deficits similar to those observed in PD [25]. The animals were treated with either vehicle, lenalidomide, or thalidomide. We used thalidomide for comparison purposes because it is the parent molecule of lenalidomide and, although it penetrates better the blood–brain barrier (BBB) [29, 30], it has lower anti-TNFα potency than lenalidomide [31, 32]. We measured total activity and performed motor behavior assessment using the round beam test (Fig. 1). The tg animals showed an increase in activity and motor errors, as previously reported for this tg model [33, 34]. Lenalidomide, but not thalidomide, reduced total activity, increased speed, and reduced the number of errors in the round beam test (Fig. 1). These results suggest that lenalidomide ameliorates behavioral deficits in a tg mouse model of PD, an ability not shared with its parent molecule thalidomide.

**Fig. 2:**
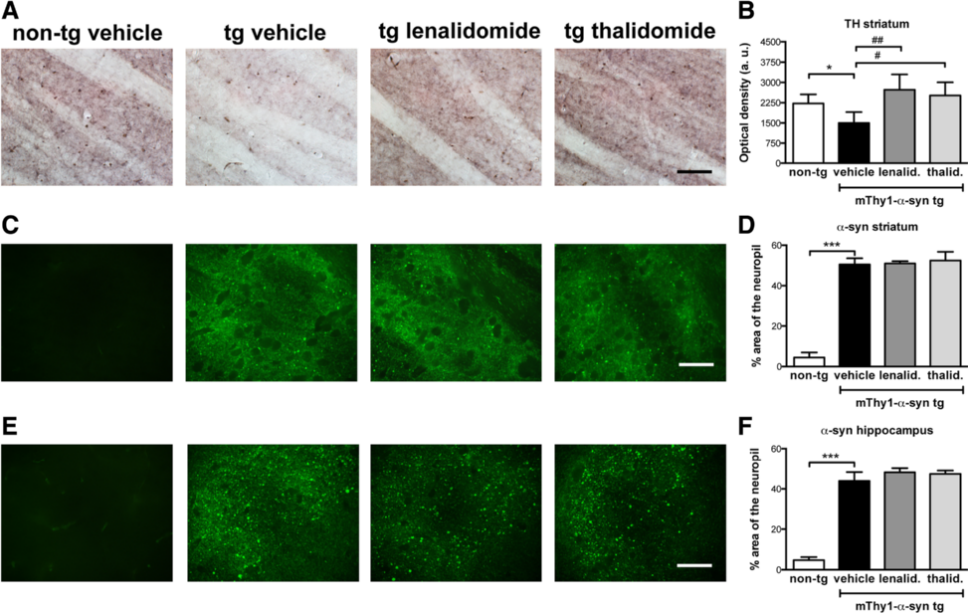
Lenalidomide reduces dopaminergic fiber loss in the striatum of mThy1-α-syn tg mice. **a** TH immunostaining of the striatum of non-tg and mThy1-α-syn tg mice treated with vehicle, lenalidomide, or thalidomide. **b** Optical density quantification of TH staining in the striatum. **c** α-syn immunostaining of the striatum of non-tg and mThy1-α-syn tg mice treated with vehicle, lenalidomide, or thalidomide. **d** Percentage of neuropil area positive for α-syn in the striatum. **e** α-syn immunostaining of the hippocampus of non-tg and mThy1-α-syn tg mice treated with vehicle, lenalidomide, or thalidomide. **f** Percentage of neuropil area positive for α-syn in the hippocampus. Error bars represent ± SEM. *, #*p* < 0.05; ##*p* < 0.01; ***p < 0.001. Scale bar = 50 μm

In PD, the parkinsonian features have been correlated to the loss of dopaminergic input to the striatum [35]. Similarly, in mThy1-α-syn tg mice, there is a reported loss of TH immunoreactive fibers in the striatum when compared to the non-tg mice (Fig. 2a, b) [33, 34]. Lenalidomide and thalidomide both restored TH immunoreactivity in the striatum of the mThy1-α-syn tg mice to levels similar to non-tg mice. Furthermore, the mThy1-α-syn tg mice show α-syn accumulation in neuronal bodies and neuropil of striatum, hippocampus (Fig. 2c–f), and other brain regions (not shown). Immunohistochemical analysis revealed that lenalidomide and thalidomide had no effect on α-syn accumulation in the mThy1-α-syn tg mice. Therefore, these results suggest that the behavioral improvement observed with lenalidomide is not a consequence of reduced α-syn accumulation.

### Lenalidomide reduced microgliosis in the mThy1-α-syn tg mice

**Fig. 3:**
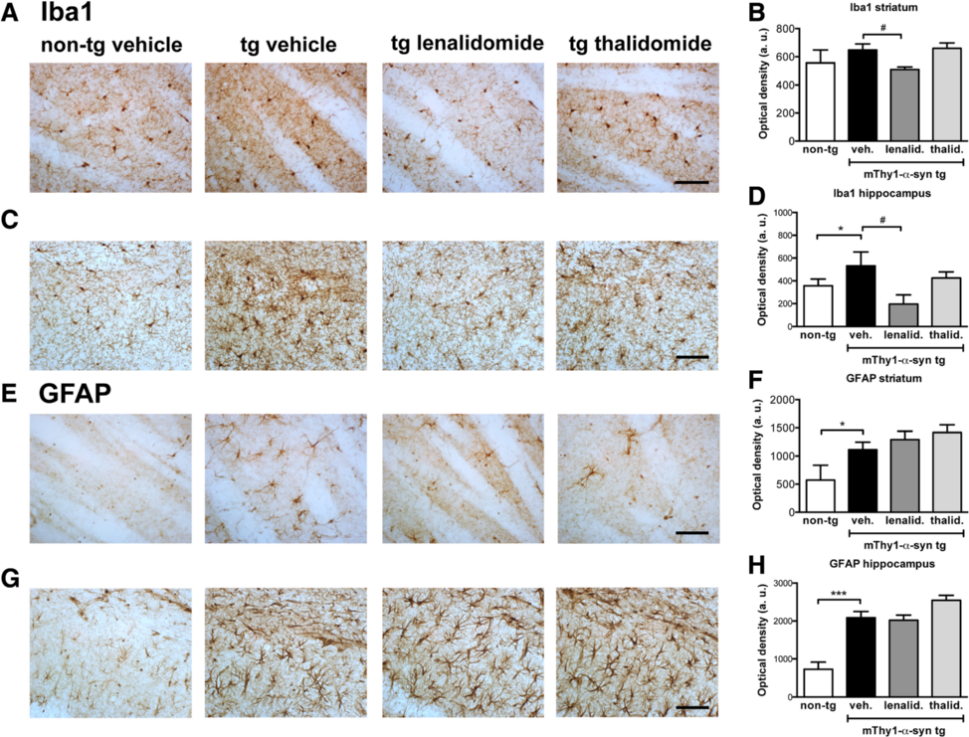
Lenalidomide reduces microgliosis in mThy1-α-syn tg mice. **a** Iba1 immunostaining of the striatum of non-tg and mThy1-α-syn tg mice treated with vehicle, lenalidomide, or thalidomide. **b** Optical density quantification of Iba1 staining in the striatum. **c** Iba1 immunostaining of the hippocampus of non-tg and mThy1-α-syn tg mice treated with vehicle, lenalidomide, or thalidomide. **d** Optical density quantification of Iba1 staining in the hippocampus. **e** GFAP immunostaining of the striatum of non-tg and mThy1-α-syn tg mice treated with vehicle, lenalidomide, or thalidomide. **f** Optical density quantification of GFAP staining in the striatum. **g** GFAP immunostaining of the hippocampus of non-tg and mThy1-α-syn tg mice treated with vehicle, lenalidomide, or thalidomide. **h** Optical density quantification of GFAP staining in the hippocampus. Error bars represent ± SEM. *, #*p* < 0.05; ***p < 0.001. Scale bar = 50 μm

Next, we analyzed glial inflammatory responses by immunostaining against the glial markers GFAP (astrocytes) and Iba1 (microglia) (Fig. 3). The tg mice showed glial reactivity in hippocampus and other brain areas when compared with non-tg controls [33], observed as an increment in cell projections and intensity of the staining. However, although there was an increase in microglial reactivity in the striatum of tg animals (Fig. 3b), the difference with non-tg animals was not statistically significant at this age. Treatment with lenalidomide or thalidomide did not affect GFAP staining. However, treatment with lenalidomide, but not thalidomide, reduced Iba1 immunoreactivity both in hippocampus and striatum (Fig. 3b, d). This amelioration of microgliosis was not observed in other brain areas (not shown). These results suggest that lenalidomide is more effective that thalidomide at reducing microglial activation.

### Lenalidomide regulated cytokine expression and NF-κB activation in the mThy1-α-syn tg mice

**Fig. 4:**
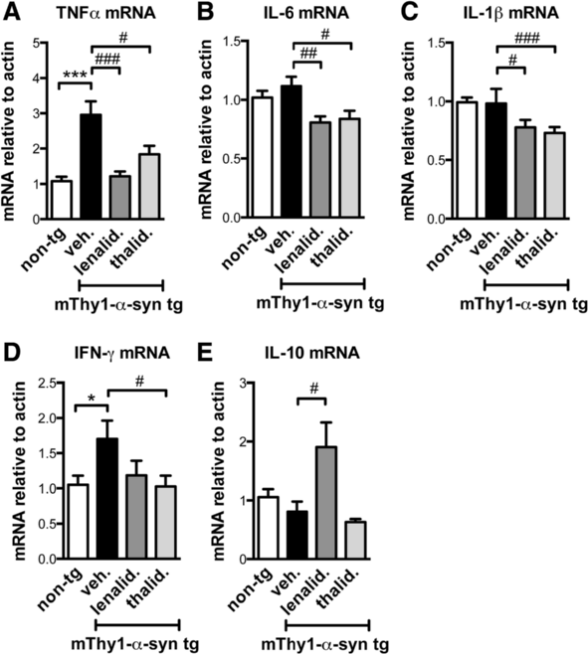
Lenalidomide modulates cytokine mRNA levels in mThy1-α-syn tg mice. The expression of relevant cytokines was measured by qPCR in non-tg and mThy1-α-syn tg mice treated with vehicle, lenalidomide, or thalidomide. **a** TNFα. **b** IL-6. **c** IL-1β. **d** IFN-γ. **e** IL-10. Error bars represent ± SEM. *, #*p* < 0.05; ##*p* < 0.01; ***, ###*p* < 0.001

Lenalidomide and thalidomide reportedly reduce the expression of levels of pro-inflammatory cytokines, such as TNFα, interferon γ (IFNγ), IL-1β, and IL-6 in peripheral blood mononuclear cells [24]. Therefore, the mice were analyzed for the expression of the most relevant pro-inflammatory and anti-inflammatory cytokines by qPCR (Fig. 4). The mThy1-α-syn tg mice showed a significant increase in TNFα mRNA levels, and both lenalidomide and thalidomide reduced TNFα expression (Fig. 4a). Lenalidomide and thalidomide also reduced IL-6, IL-1β, and IFNγ expression (Fig. 4b–d). Interestingly, lenalidomide also increased the expression of the anti-inflammatory cytokine IL-10, while thalidomide did not (Fig. 4e). These results suggest that lenalidomide might have better anti-inflammatory properties than thalidomide in the tg mouse brain.

**Fig. 5:**
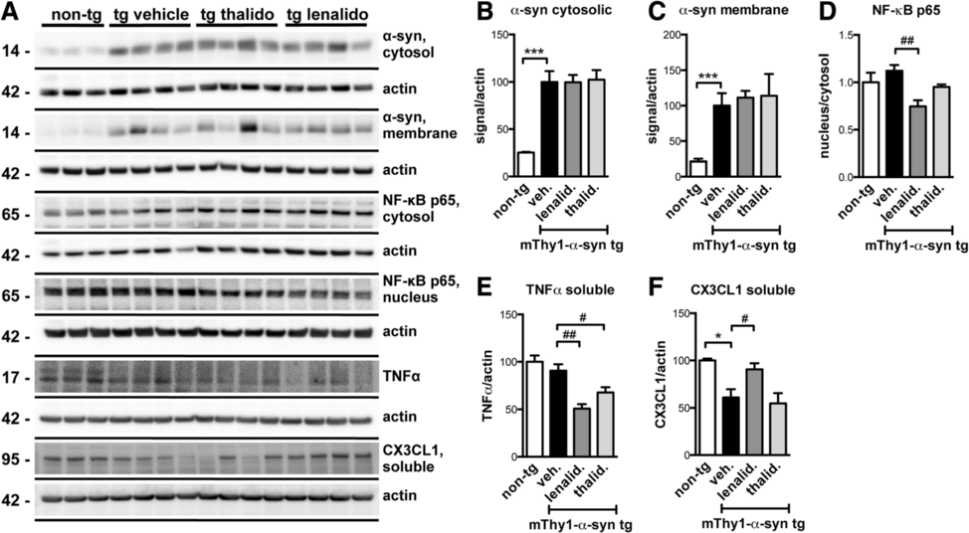
Lenalidomide modulates NF-κB signaling and cytokine expression in mThy1-α-syn tg mice. **a** Immunoblot analysis of α-syn, NF-κB p65, TNFα, and CX3CL1 (fractalkine) in protein extracts from non-tg and mThy1-α-syn tg mice treated with vehicle, lenalidomide, or thalidomide. Significant results of three to four mice per group are shown. **b** Densitometric analysis of the levels of the α-syn immunoreactive band in cytosol, normalized by actin levels. **c** Densitometric analysis of the levels of the α-syn immunoreactive band in the particulate/membrane fraction, normalized by actin levels. **d** Densitometric analysis of the levels of the NF-κB p65 immunoreactive band, expressed as the ratio between the nuclear and cytosolic fractions. **e** Densitometric analysis of the levels of the TNFα immunoreactive band in cytosol, normalized by actin levels. **f** Densitometric analysis of the levels of the fractalkine immunoreactive band in cytosol, normalized by actin levels. Error bars represent ± SEM. *, #*p* < 0.05; ##*p* < 0.01; ***p < 0.001

Immunoblot analysis of α-syn levels in cytosolic and particulate (membrane) fractions revealed that treatment with lenalidomide or thalidomide did not modify α-syn levels (Fig. 5a–c). NF-κB activation, measured as the ratio between nuclear and cytosolic fractions, showed that lenalidomide significantly inhibited NF-κB signaling (Fig. 5d), which is consistent with its proposed mechanism of action. TNFα protein levels were not significantly elevated in tg animals when compared to non-tg controls, but both drugs were able to reduce basal TNFα levels (Fig. 5e). Finally, CX3CL1 (fractalkine) levels were reduced in the tg mice and increased in the lenalidomide-treated mice (Fig. 5f). Fractalkine has been proposed to mediate neuron-microglial communication in the central nervous system (CNS), due to the fact that it is expressed by neurons and its receptor is expressed by microglia [36, 37]. Fractalkine expression is regulated by NF-κB [38], and it is involved in microglial activity with neuroprotective properties [39], further supporting the idea that lenalidomide has anti-inflammatory effects *in vivo*.

**Fig. 6:**
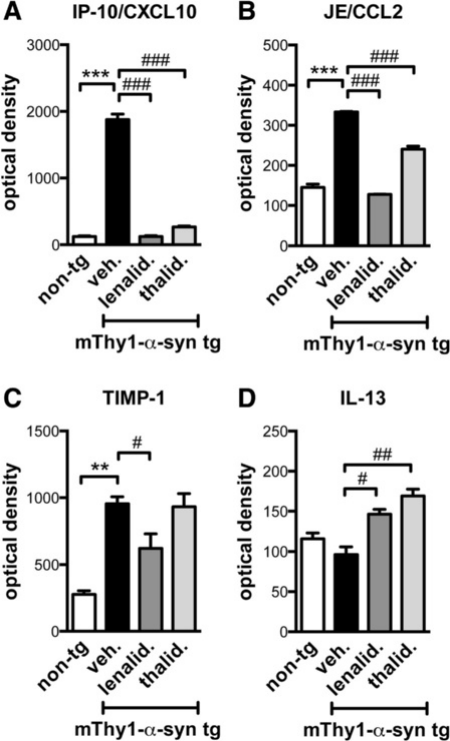
Lenalidomide modulates pro-inflammatory and anti-inflammatory cytokine protein levels in mThy1-α-syn tg mice. Additional cytokine levels were analyzed using a proteomic array in the cytosolic fraction of non-tg or mThy1-α-syn tg mice treated with vehicle, lenalidomide, or thalidomide. Significant results are expressed as relative optical density relative to the non-tg vehicle condition. **a** IP-10/CXCL10. **b** JE/CCL2. **c** TIMP-1. **d** IL-13. Error bars represent ± SEM. #*p* < 0.05; **, ##*p* < 0.01; ***, ###*p* < 0.001

Immunoblot results were complemented utilizing a protein array that measures the relative changes of 40 cytokines and chemokines (Fig. 6 and Additional file 1). Using this array we identified IP-10/CXCL10, JE/CCL2, and TIMP-1 as pro-inflammatory cytokines that were significantly dysregulated in the tg mice and which levels were partially or totally restored after lenalidomide treatment. Moreover, levels of the anti-inflammatory cytokine IL-13 were elevated after lenalidomide treatment. These results confirm the anti-inflammatory properties of lenalidomide in this tg mouse model.

### Lenalidomide regulated microglial NF-κB activation and TNFα levels *in vitro*

**Fig. 7:**
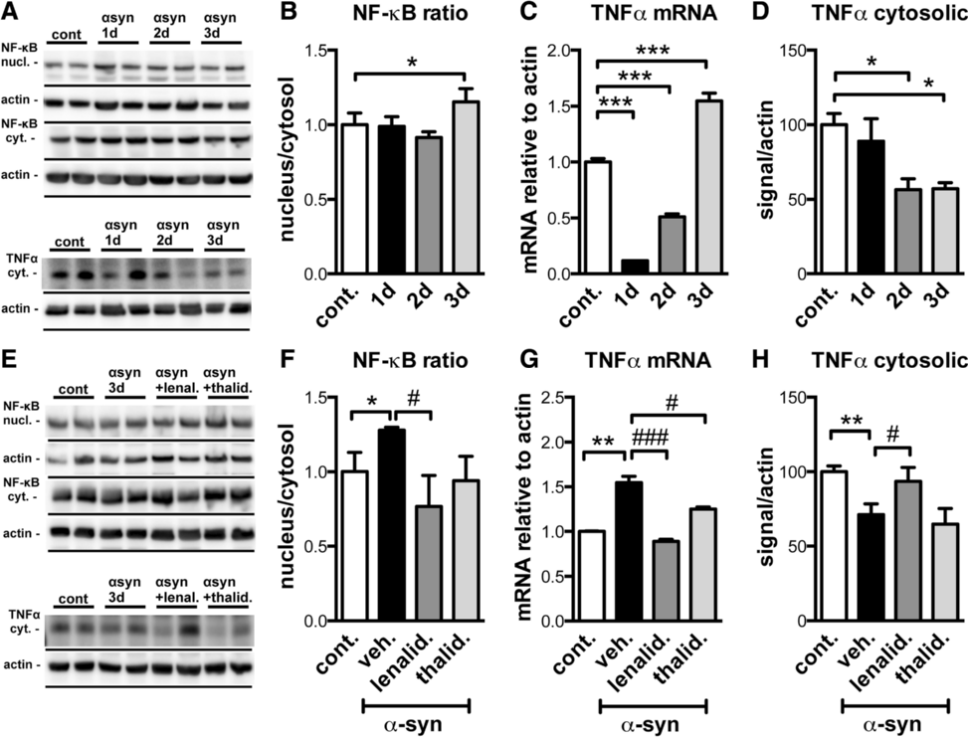
Lenalidomide inhibits NF-κB signaling and normalizes TNFα mRNA and protein levels in α-syn-treated microglial cells *in vitro*. **a** Microglial BV-2 cells were challenged with oligomeric recombinant human α-syn 5 μM for 1, 2, or 3 days, and protein extracts analyzed by immunoblot. Significant results of two independent samples per condition are shown. **b** Densitometric analysis of the levels of the NF-κB p65 immunoreactive band, expressed as the ratio between the nuclear and cytosolic fractions after the time course with α-syn. **c** qPCR analysis of the levels of TNFα mRNA after the time course with α-syn. **d** Densitometric analysis of the levels of the cytosolic TNFα immunoreactive band after the time course with α-syn. **e** BV-2 cells were challenged with oligomeric recombinant human α-syn 5 μM ± lenalidomide or thalidomide 250 μg/ml for 3 days, and protein extracts analyzed by immunoblot. Significant results of two independent samples per condition are shown. **f** Densitometric analysis of the levels of the NF-κB p65 immunoreactive band, expressed as the ratio between the nuclear and cytosolic fractions after 3 days of treatment. **g** qPCR analysis of the levels of TNFα mRNA after 3 days of treatment. **h** Densitometric analysis of the levels of the cytosolic TNFα immunoreactive band after 3 days of treatment. Error bars represent ± SEM. *, #*p* < 0.05

In order to confirm the results obtained *in vivo* in the mThy1-α-syn tg mice, and to further investigate the effects of lenalidomide in microglial cells, we used the mouse microglial cell line BV-2 [27]. BV-2 cells were first incubated with recombinant oligomeric α-syn 5 μM in a time course analysis for 1, 2, and 3 days, and the activation of NF-κB and intracellular TNFα levels were measured by immunoblot (Fig. 7a–d). The expression of TNFα was further analyzed by qPCR. After 3 days of treatment, NF-κB activation, measured as the NF-κB p65 nucleus/cytosol ratio, was significantly induced by the treatment. The levels of TNFα mRNA decreased after 1–2 days of treatment with α-syn; however, after that initial reduction, TNFα mRNA levels raised significantly above control levels after 3 days of treatment. Interestingly, intracellular TNFα levels significantly decreased after 2 days of incubation, probably as a consequence of its release to the extracellular medium, due to different turnover or transduction/translation rates, or a combination of those factors.

Next, BV-2 cells were treated with α-syn 5 μM plus lenalidomide or thalidomide at 250 μg/ml for 3 days (Fig. 7e–h). Lenalidomide inhibited NF-κB translocation to the nucleus and normalized TNFα mRNA (*p* < 0.001) and protein to levels similar to the control condition. Thalidomide did inhibit NF-κB translocation (albeit not significantly) and reduced TNFα mRNA levels (*p* < 0.05) but had no effect on the intracellular levels of this protein. These results confirm that lenalidomide is more potent than thalidomide at reducing TNFα expression in microglial cells and suggest that this increased potency may account for some of the differences observed between both molecules *in vitro* and *in vivo*.

In summary, our results suggest that lenalidomide is more effective than thalidomide at reducing neuroinflammation in the mThy1-α-syn tg model of PD. Furthermore, lenalidomide or similar thalidomide derivatives might be of use for ameliorating maladaptive neuroinflammation in PD and other synucleinopathies.

## Discussion

In this manuscript, we investigate the ability of lenalidomide at reducing neuroinflammatory responses in a tg mouse model of PD. Lenalidomide ameliorated motor behavioral deficits, protected from dopaminergic fiber loss, reduced pro-inflammatory cytokine levels, and inhibited NF-κB activation *in vivo* and *in vitro*. Its parent molecule thalidomide, used for comparative purposes, normalized some but not all of these parameters. Our results suggest that lenalidomide or related immunomodulatory drugs might be good candidates for reducing neuroinflammation in PD or related synucleinopathies.

Recent studies support the notion that inflammation plays an important role in the pathogenesis of PD [40]. The mechanisms are not completely understood; however, factors triggering inflammation may include dysregulation of inflammatory pathways, pathogens, environmental toxins, and α-syn aggregates [40]. We have recently shown that aggregated toxic α-syn released from affected neurons can transmit to other neurons and glial cells [15, 41], leading to activation of TLR-dependent pathways in microglia and astrocytes. It has been suggested that activation of TLRs might lead to NF-κB translocation to the nucleus and expression of pro-inflammatory genes [42], and evidence supports a role for signaling downstream of TLRs in PD [43, 19, 44, 45]. Therefore, in addition to its direct toxic effects in neurons, α-syn could induce neurodegeneration by dysregulating pro-inflammatory pathways. Although physiological activation of inflammatory responses might play a beneficial role in clearing moderate levels of extracellular α-syn, an over-activation of these responses (maladaptive inflammation) could result in neurodegeneration [46]. Supporting a role for maladaptive inflammation in PD, epidemiological studies have shown that ibuprofen use is associated with decreased PD risk [47, 48], and anti-inflammatories reduce degeneration of dopaminergic neurons in models of PD [49]. Moreover, in PD and animal models, activated microglia and astroglia with increased TNFα have been described [17].

The mThy1-α-syn tg mouse model [50] displays microgliosis, astrogliosis, and dysregulation of pro-inflammatory pathways with TLR activation [18]. As neuroinflammatory cascades triggered by α-syn aggregates might involve NF-κB activation [51, 52], it follows that targeting this pathway might be of therapeutic value. Here, we show that the immunomodulatory drug lenalidomide attenuates the pathology in a α-syn tg model of PD. Lenalidomide is a small thalidomide derivative with anti-angiogenic and immunomodulatory activity that has shown therapeutic effects in multiple myeloma [53–55] and in animal models of amyotrophic lateral sclerosis [56, 57]. Its mechanisms of action involve T cell co-stimulation [58–60], increased NK cell proliferation and function [58, 60], and inhibition of the production of TNFα [61, 55, 60], IL-1β, IL-6, IL-12, and IFNγ [24]. At the molecular level, lenalidomide has been shown to inhibit NF-κB signaling [21], as confirmed by our results. Interestingly, lenalidomide compensates for lower BBB penetration by displaying higher anti-TNFα and anti-inflammatory potency than thalidomide [29, 30, 62, 31, 32]. Our results support this observation; however, more in-depth studies of BBB penetration should be performed in the future. Interestingly, TNFα protein levels were not significantly elevated in tg animals when compared to non-tg controls, suggesting a higher rate of mRNA transcription compared to protein translation and/or different turnover rate. However, we cannot rule out the possibility of local and/or cell-specific changes in TNFα protein levels that may be functionally relevant. Finally, regarding the different anti-inflammatory properties observed between lenalidomide and thalidomide, it can also be suggested that different cell types and/or brain regions may be more sensitive to lenalidomide than to thalidomide, and we cannot rule out the possibility that thalidomide may exert additional detrimental effects that could lower its global anti-inflammatory properties compared to lenalidomide. Indeed, the use of derivatives such as lenalidomide might circumvent the potential secondary effects of thalidomide [63, 64]. Nevertheless, to take full advantage of the therapeutic potential of lenalidomide, a risk assessment for neurotoxicity would be recommended on a case-by-case basis.

Our results show that lenalidomide improved behavioral deficits in the mThy1-α-syn tg mice. This improvement was associated to reduced dopaminergic fiber loss and reduced microgliosis. The observation that lenalidomide is more effective at improving behavioral deficits than thalidomide may be related to the fact that it reduces microgliosis and pro-inflammatory cytokine production more effectively than its parent molecule, parameters that are usually associated with more severe behavioral deficits in tg models of PD. Interestingly, the effect of lenalidomide was specific to microglial cells and it did not affect astrogliosis. Differences in cell-specific expression profiles of pro-inflammatory cytokines might be an explanation for this observation. In this sense, it is also important to note that lenalidomide acts as a potent immunomodulator in macrophages [65, 58]. Indeed, our *in vitro* results confirm that lenalidomide inhibits NF-κB signaling and normalizes TNFα mRNA and protein levels in a microglial cell line. Nevertheless, in order to accurately identify its target cells in CNS, a more extensive analysis using different cell types will be necessary.

Lenalidomide also normalized levels of other cytokines such as fractalkine, IP-10, JE, and TIMP-1. Fractalkine is constitutively expressed by neurons and is able to reduce microglial activity by binding to its receptor on glial cells [39]. In a model of PD, the lack of fractalkine receptor results in exacerbation of neuronal cell death and increased production and release of IL-1β by microglia [39]. Levels of IP-10 correlate with cognitive status in PD patients [66], and levels of TIMP-1, an endogenous tissue inhibitor of matrix metalloproteinases, are significantly elevated in substantia nigra in PD [67]. Finally, it has been observed that JE levels were upregulated in the striatum and the ventral midbrain of a model of PD [68]. The fact that lenalidomide is able to modulate the expression of these cytokines further confirms its anti-inflammatory activity in the mThy1-α-syn tg mice.

## Conclusions

In conclusion, lenalidomide is able to reduce motor deficits, dopaminergic fiber loss, microgliosis, and pro-inflammatory cytokine expression in a tg mouse model of PD. At the molecular level, lenalidomide inhibits NF-κB activation and TNFα expression both *in vivo* and *in vitro*. These results suggest that lenalidomide or similar immunomodulatory compounds might be of use for the management of neuroinflammation in PD and related disorders.

## Additional file

Additional file 1:
**Changes in cytokine and chemokine levels in a transgenic model of PD.** Cytokine levels in the cytosolic fraction of mThy1-α-syn transgenic mice treated with vehicle, lenalidomide, or thalidomide were analyzed using a proteomic array. Results indicate the percentage of increase (+) or decrease (−) of the signal with respect to non-tg control values. **p* < 0.05; ***p* < 0.01; ****p* < 0.001.

## Abbreviations

ANOVA: Analysis of variance
α-syn: Alpha-synuclein
BBB: Blood–brain barrier
CNS: Central nervous system
GFAP: Glial fibrillary acidic protein
Iba1: ionized calcium-binding adapter molecule 1
IFNγ: Interferon γ
IL: Interleukin
PD: Parkinson’s disease
qPCR: Quantitative real-time polymerase chain reaction
tg: Transgenic
TH: Tyrosine hydroxylase
TLR: Toll-like receptor
TNFα: Tumor necrosis factor α
