# Supplementary material for: Lenalidomide reduces microglial activation and behavioral deficits in a transgenic model of Parkinson’s disease
Source: J Neuroinflammation. 2015 May 14;12:93. doi: 10.1186/s12974-015-0320-x (PMC4432827; doi:10.1186/s12974-015-0320-x)
Supplement: Additional file 1: — Changes in cytokine and chemokine levels in a transgenic model of PD. Cytokine levels in the cytosolic fraction of mThy1-α-syn transgenic mice treated with vehicle, lenalidomide, or thalidomide were analyzed using a proteomic array. Results indicate the percentage of increase (+) or decrease (−) of the signal with respect to non-tg control values. *p < 0.05; **p < 0.01; ***p < 0.001. [file 12974_2015_320_MOESM1_ESM.docx]

|  |  | **mThy1-α-syn** | | |
| --- | --- | --- | --- | --- |
| **Target** | **Alternate name** | **tg vehicle** | **tg lenalidomide** | **tg thalidomide** |
| **IL-1α** | **IL-1F1** | -2.40 | -17.88** | -6.16 |
| **IL-1β** | **IL-1F2** | +17.35 | -27.48 | +13.08 |
| **IL-1ra** | **IL-1F3** | +105.92* | -7.43 | +121.38* |
| **IL-2** |  | +4.38 | -0.28 | +26.65 |
| **IL-3** |  | +44.90* | -13.48 | -2.24 |
| **IL-4** |  | +8.50 | -13.40 | -20.27 |
| **IL-5** |  | -31.92 | -44.08 | -22.74 |
| **IL-6** |  | +14.10 | -46.59 | +12.99 |
| **IL-7** |  | -30.81** | -21.01* | -13.94 |
| **IL-10** |  | -6.97 | -9.94 | +14.33 |
| **IL-13** |  | -16.78 | +26.23 | +45.82* |
| **IL-12 p70** |  | -24.72 | +14.51 | +32.21 |
| **IL-16** |  | +4.94 | -14.41* | +13.41 |
| **IL-17** |  | -36.03 | -10.09 | -14.41 |
| **IL-23** |  | -15.10 | +1.01 | +23.06 |
| **IL-27** |  | -20.12 | -6.17 | +7.59 |
| **I-309** | **CCL1/TCA-3** | -28.25* | -43.09** | +6.71 |
| **JE** | **CCL2/MCP-1** | +129.37*** | -12.17 | +65.54*** |
| **MIP-1α** | **CCL3** | -26.65 | +28.29 | -5.46 |
| **MIP-1β** | **CCL4** | -5.18 | +13.12 | +8.68 |
| **RANTES** | **CCL5** | -14.92 | +48.92 | +13.93 |
| **Eotaxin** | **CCL11** | -22.08 | -44.37** | -1.28 |
| **MCP-5** | **CCL12** | -6.87 | +2.60 | +26.20* |
| **TARC** | **CCL17** | +45.97* | +73.21** | +23.20 |
| **KC** | **CXCL1** | +27.07 | -3.93 | +12.90 |
| **MIP-2** | **CXCL2** | -0.76 | +29.17 | +14.03 |
| **MIG** | **CXCL9** | -3.16 | +60.70** | +32.49* |
| **IP-10** | **CXCL10/CRG-2** | +1425.16*** | +0.32 | +117.45 |
| **I-TAC** | **CXCL11** | +11.98 | -2.09 | -19.14 |
| **SDF-1** | **CXCL12** | -3.19 | +17.72 | +13.59 |
| **BLC** | **CXCL13/BCA-1** | +24.46* | -23.03* | -22.71 |
| **C5/C5a** |  | +31.18 | -12.05 | -34.00 |
| **G-CSF** |  | +78.28 | -34.04 | -18.02 |
| **GM-CSF** |  | +3.60 | -34.36** | +6.15 |
| **M-CSF** |  | +15.65* | -3.56 | +3.46 |
| **sICAM-1** | **CD54** | +1.21 | -9.32 | +30.67*** |
| **IFN-γ** |  | -21.51 | -7.12 | +20.68 |
| **TNF-α** |  | +4.62 | -27.03* | -26.37* |
| **TIMP-1** |  | +244.24** | +124.11 | +236.27** |
| **TREM-1** |  | -21.88 | +59.88 | +2.84 |

**Additional File 1. Changes in cytokine and chemokine levels in a transgenic model of PD.** Cytokine levels in the cytosolic fraction of mThy1-α-syn transgenic mice treated with vehicle, lenalidomide or thalidomide were analyzed using a proteomic array. Results indicate the percentage of increase (+) or decrease (-) of the signal with respect to non-tg control values. * p<0.05, ** p<0.01. *** p<0.001.
